# Evaluation and Modification of the 10‐Item Short Form Communicative Participation Item Bank for Persons with Primary Progressive Aphasia and their Communication Partners

**DOI:** 10.1002/alz.091754

**Published:** 2025-01-09

**Authors:** Ollie Fegter, Sara Shaunfield, Matthew Bona, Angela C Roberts, Emily J Rogalski

**Affiliations:** ^1^ Northwestern University Feinberg School of Medicine, Chicago, IL USA; ^2^ University of Chicago, Chicago, IL USA; ^3^ Western University, London, ON Canada

## Abstract

**Background:**

Primary progressive aphasia (PPA) is a dementia syndrome characterized by language and communication impairments, with relative sparing of other cognitive domains. As a relatively rare dementia syndrome, there are few measures developed and validated for individuals with PPA. Development of outcome measures tailored to the communication experiences of persons with PPA (PwPPA) is critical for the accurate assessment of interventions success. The 10‐item Communicative Participation Item Bank (CPIB) has been used as a measure for communication participation in PPA. However, the CPIB was validated as a disorder‐agnostic measure and its content validity for PPA has not been evaluated. This study aims to evaluate the face and content validity of the 10‐item CPIB for PwPPA and their communication partners (CP).

**Method:**

Cognitive interviews were conducted with participants (N = 12) who had previously completed the CPIB during the Communication Bridge 2 randomized controlled trial (NCT03371706) and with four participants unfamiliar with the CPIB. PwPPAs and CPs completed the CPIB during a semi‐structured interview assessing measure format, instructions, response options, item comprehension, and item relevance. To evaluate content validity, participants were asked open‐ended questions to elicit relevant communication participation experiences missing from the questionnaire. Closed‐ended responses (e.g., clarity, relevance) were tabulated, and open‐ended responses (e.g., item comprehension, missing content) were thematically analyzed. Summaries of measure format, instructions, and response options were generated, as well as item‐level findings regarding comprehension, relevance, and missing content.

**Result:**

Both PwPPAs and CPs: a) considered the instructions to be clear (n = 16, 100%); b) recommended adding a fifth response option (e.g., “Somewhat; n = 11) to better represent the range of communication situations; c) reported all items except one (“persuade…to see a different point of view”) were relevant (n≥13; ≥81%, for 9 items); and d) talking on the phone was the most reported missing communicative participation situation (n = 12; 75%). Further, 3 participants reported talking over videochat, and 4 participants reported email/texting as missing. Results were similar between CPIB‐experienced and CPIB‐naïve participants.

**Conclusion:**

Findings indicate that modifications to the 10‐item CPIB short form may be needed for use with PwPPA and their CPs to more fulsomely capture communication participation in PPA.

